# Optimisation of T2 and T2* sequences in MRI for better quantification of iron on transfused dependent sickle cell patients

**DOI:** 10.1038/s41598-021-88116-8

**Published:** 2021-04-19

**Authors:** Azza Ahmed, Amani Baldo, A. Sulieman, Hind Mirghani, Fouad A. Abolaban, I. I. Suliman, Isam Salih

**Affiliations:** 1 Sudan Atomic Energy Commission, Al Gamah Street, P. O. Box 3001, Khartoum, Sudan; 2 Jaffar Bin Auf Paediatric Hospital, Hosptials Road, Khartoum, Sudan; 3 Radiology and Medical Imaging Department, College of Applied Medical Sciences, Prince Sattam Bin Abdulaziz University, P. O. Box 422, Alkharj, 11942 Saudi Arabia; 4 Nuclear Engineering Department, Faculty of Engineering, King Abdulaziz University, P. O. Box 80221, Jeddah, 21589 Saudi Arabia; 5 Physics Department, College of Science, Imam Mohammad Ibn Saud Islamic University (IMSIU), P. O. Box 11642, Riyadh, Saudi Arabia; 6 Basic Science Department, Prince Sattam Bin Abdulaziz University, P. O. Box 422, Alkharj, 11942 Saudi Arabia

**Keywords:** Magnetic resonance imaging, Sickle cell disease, Imaging techniques, Paediatric research

## Abstract

This work aimed to investigate the effect of different shim techniques, voxel sizes, and repetition time (TR) on using theT2 and T2* sequences to determine their optimum settings to investigate the quantification of iron in transfused dependent sickle cell patients. The effect of each of these parameters was investigated on phantoms of different Gadolinium (Gd) concentrations, on 10 volunteers and 25 patients using a1 5T MRI Philips scanner. No significant difference between the three shim techniques was noticed in either T2 or T2* sequence measurements. Pixel sizes of 1 × 1 and 2 × 2 mm provided optimum results for T2 measurements. At 1 × 1 mm pixel size the T2* measurements experienced less error in measurements than the size of 2.5 × 2.5 mm used in the literature. Even though the slice thickness variation did not provide any changes in T2 measurements, the 12 mm provided optimum T2* measurements. TR variation did not yield significant changes on either T2 or T2* measurements. These results indicate that both T2 and T2* sequences can be further improved by providing more reliable measurements and reducing acquisition time.

## Introduction

Sickle cell anaemia (SCA) is a haemoglobinopathy caused from a single point mutation in the β-chain of human haemoglobin^1^ It causes rigid and sickle-shaped red blood cells, eventually ends up in anaemia, which leads to multiple complications, the most serious of which is the Overt stroke^2^.

In Africa, sickle cell disease is raising great health concerns, particularly in the western part of Sudan among the Baggara (Misseriya tribe). Archibald^3^ stated that the first incidence of sickle cell in Sudan was reported in 1926. The number of cases of this disease reached 30% among pastoral tribes in western Sudan. Up to 16% was found in immigrant tribes in the area^4^.

The prevalence of patients with SCA in Sub-Saharan Africa ranges between 5 and 40%^5^. Their life expectancy is < 20 years old^6^, and patients younger than 5 years are at high risk of death.

Blood transfusions in sickle cell patients could lead to iron overload particularly in the liver. Iron overload promotes the formation of toxic oxygen radicals that can cause cell damage. Therefore, the knowledge of iron levels in these patients is important to mitigate the health consequences of iron overload.

The levels of iron in blood-transfusion patients are often monitored using the serum ferritin method. This method known to be an unreliable marker for assessing iron in organs such as the heart and liver^7^.

The use of magnetic resonance imaging (MRI) techniques involving transverse relaxation times such as the T2 and T2* have shown promising results in estimating iron concentration in transfusion-dependent patients^8,9^. It is therefore, expected that the serum ferritin and invasive biopsy methods will be replaced by the MRI methods, however, there is limited data in the literature that systematically compared T2 and T2* sequences in sickle cell anemia (SCA)^10^. For the quantification of iron using MRI techniques, the literature highlighted that MR signal measurements could be affected by sequence protocol^11–14^. Nevertheless, many of these studies were limited to narrow investigations of parameter effects on T2 and T2* measurements. Researchers stated that T2 techniques are not affected by the size or shape of the imaging voxel^15^ or shimming techniques^16^, and commented that T2* methods were affected by voxel size^17^, shimming technique and TR.

The empirical effects of shimming techniques, range of voxel sizes, and TRs were not been sufficiently investigated. There is also a lack of understanding of how these changes in parameters affect T2 or T2* measurements. Reported T2 and T2* values in healthy subjects showed large variation between centres which could be explained due to the acquisition standardisation^18^. While the literature highlighted some guidelines for use of protocols for T2* measurements for cardiovascular image^19^, different acquisition approaches were implemented for T2 sequences and hence lead to variations in T2 values^20^. It was also suggested that changing T2* sequence parameters may particularly be significant at high liver concentrations^9^.

Gradient echo sequence (T2*), and Ferriscan (T2 sequences) have been used for evaluating iron in patients with iron overload, however, Ferriscan is not widely available in Africa.

The T2 sequence is currently a validated sequence for quantification of iron, on the other hand it is technically difficult to implement and requires additional costs and delay for data analysis^21^. The T2 sequence that is available in most scanners is the Fast Spin Echo Sequence or Turbo Spin Echo (TSE) sequence, and has been suggested to be used for iron quantification in transfusion-dependent patients^22^.

Herein we thought to investigate the effect of different shim techniques, voxel sizes, and repetition times (TR) on the quantification of iron in transfusion-dependent sickle cell patients and examining optimum settings for appropriate assessment of patients.

The present study was built on the experimental hypothesis in changing settings and optimizing parameters that may lead to improving the quality of the images produced, which leads to the appropriate evaluation transfusion dependent patients.

## Materials and method

The study was conducted at Dar-Alateeba Hospital, a private hospital located in Khartoum State, Sudan. The measurements involved the use of a 1.5 T whole-body Philips-MRI scanner. The scanner was equipped with high performance gradients with a maximum strength of 40 mT/m and a maximum slew rate of 200 T/m/s. A four-element torso phased array coil was used for all the scans. The research work explored the best settings that can be used in patients’ measurement via investigations conducted first on a phantom, on healthy volunteers and finally on patients as described in the following subsections.

### Phantom study and calibration

Phantom was used in the study to simulate the relaxation properties of tissues with different concentrations of iron. It consisted of a Plexiglas holder containing eight plastic bottles, each with a volume of 17 ml. Seven bottles contain gadolinium (Gd) at concentration of 0.5, 1, 2, 3, 4, 5 and 20%. One of the bottles contained pure water for reference. The effect of the paramagnetic gadolinium on the signal intensity measurements using T1, T2 and T2* sequences has been previously investigated in number of studies^23–25^. Researchers reported that the increase in gadolinium concentrations led to a significant decrease on the T2 and T2* relaxation^26,27^.

The phantom was initially scanned with a balance fast field echo (FFE) sequence to localise the best plane for analysis. Following this a Turbo Spin Echo (or T2 sequences) and a Gradient Echo (or T2* sequence) were run on the phantom first for calibration using the following parameters: shim = default, pixel size = 2 × 2 mm, slice thickness = 10 mm, TE/ES = 4.4/4.4 and TR = 400 ms, which gives an acquisition time of 27.2 s. For T2* sequence the settings were: shim = default, pixel size = 2 × 2 mm, slice thickness = 10 mm, TE/ES = 2.5/1.1 ms, and TR = 200 ms and this gives an acquisition time of 7.8 s. The number of echoes for each of the T2 and T2* sequences were 20 and 15 respectively. The scan for each of the two calibration protocols was repeated 3 times. Their mean and standard deviation (SD) of the repeat measurements were obtained.

Following this, optimization protocols were carried out for both the T2 and T2* sequences and it included the effects of the calibration parameters as well as the effects of the remaining: the shim techniques, four pixel sizes, two slice thicknesses and four TR values. For each of the imaging parameters, the scan is repeated three times on which also the mean and SD were calculated for the two sequences.

### Volunteers and patients

The results obtained from the phantom study were tested on 10 volunteers and 25 patients. The volunteers were healthy, with no known previous diseases. Initially, serum ferritin measurements were performed for all patients and volunteers. The studied subjects were scanned for the following parameters: Shim default, auto and volume for both T2 and T2* sequence. Pixel sizes were 2 × 2, 2.5 × 2.5, 3.28 × 3.28 and 4 × 4 mm for T2 and 1 × 1, 2 × 2, 2.5 × 2.5 and 3.28 × 3.28 mm for T2*. TR values were 245, 330, 400, and 500 ms for T2, and 100,160, 200, and 230 ms for T2* sequence. Slice thickness of 8, 10, and 12 mm was also investigated on the phantom for both T2 and T2* sequences.

The patients’ study was conducted at the optimum settings obtained from both the phantom and volunteers’ studies using both T2 and T2* sequences. These were then compared to the calibration/standard settings, which was implemented before the incremental changes. The settings for the optimised sequence for T2 were: shim = auto, pixel size = 2 × 2 mm, slice thickness = 10 mm, TE/ES = 4.4/4.4 ms, and TR = 245 ms, with an acquisition time = 16.9 s. The optimised settings for the T2* sequence were as follow: shim = default, pixel size = 1 × 1 mm, slice thickness = 12 mm, TE/ES = 3.7/1.7 ms and TR = 100 ms-with acquisition time = 5.8 s.

For paediatric patients not able to breath-hold, the sequence was implemented during free breathing using respiratory triggering.

All images acquired were analyzed by plotting a circular region of interest (ROI) using Matlab code (version 2015b). For the phantom study, the ROI was chosen around each of the eight bottles. As for the volunteers and patients, regions away from the vascular structure were chosen to avoid the contribution of artefacts from the blood. The pixel intensities of each of the selected region were averaged together and this region is propagated to other images with longer echo times (TE). The signals were then plotted versus their corresponding TE values using Levenberg–Marquardt algorithm of non-linear curve fitting. Data from T2 and T2* were fitted using a simple exponential decay formula^28^ (Eq. 1):

1 $${\text{S}} = {\text{S}}_{0} \exp ^{{ - {\text{TE}}/{\text{T}}2^{*} }}$$

where S is the final signal intensity, S_0_ is the initial signal and TE is the echo time.

All data points at noise level were removed using the truncation method as they are currently widely used^29^ and proven to provide more accurate measurements than the offset and baseline subtraction methods^9^.

All Statistical analysis was performed using EXCEL spreadsheets. To evaluate the differences between the two data sets, a paired Student’s *t*-test was calculated. A *p* value less than 0.05 was considered to be significant. Image analysis were carried out using a personally written programming code (Matlab 2015b).

Ethical approval was obtained from the Health Research Council-National Research Ethics Committee (Ministry of Health, Sudan). The committee was established to review ethical issues in health research proposals submitted for implementation, in the country, and review its results in accordance with regulations that adopt international guidelines. Written informed consent was obtained from each individual for carrying out the studies.

## Results and discussion

### Results

**Figure 1:**
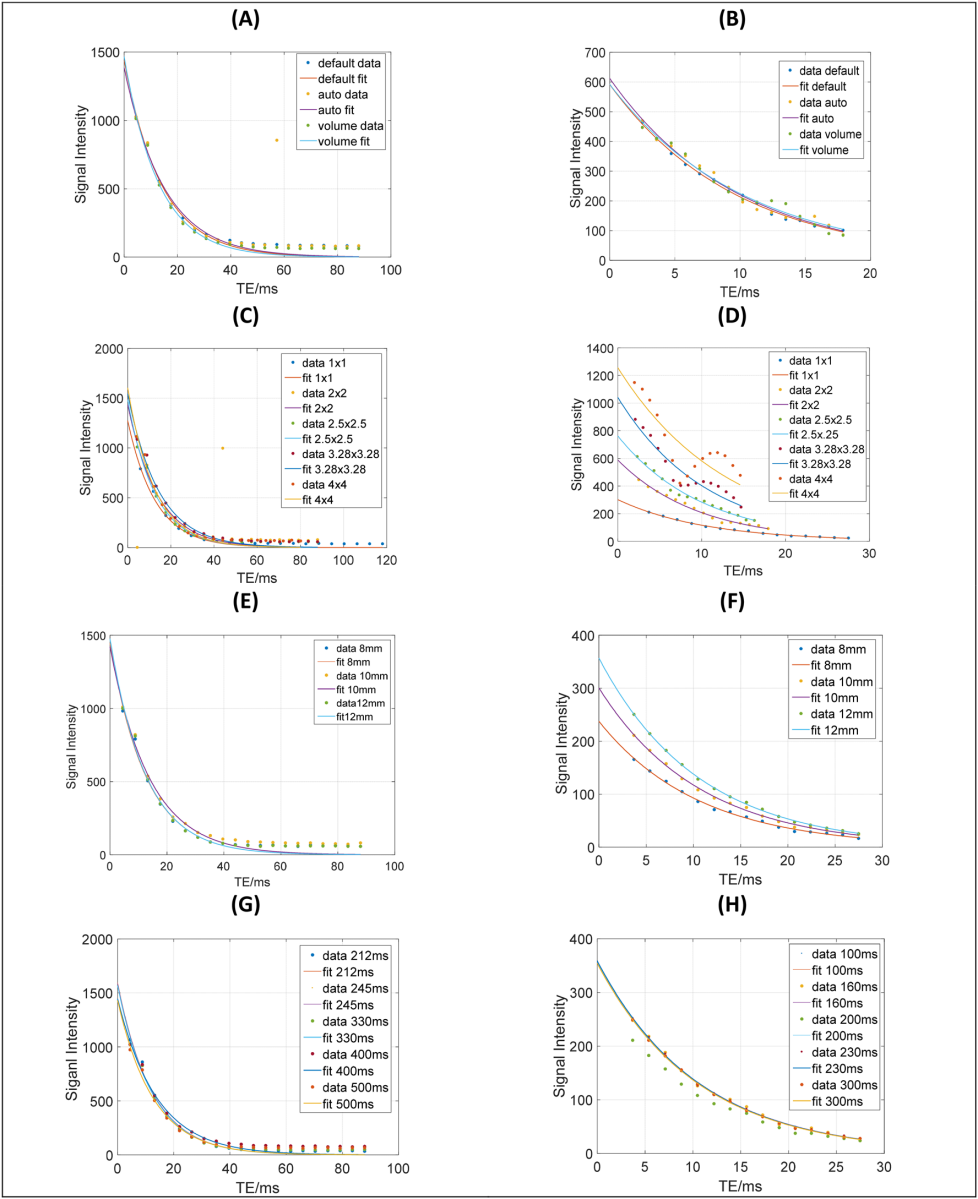
Decay curves of the 2% Gd concentration for T2 and T2* sequences, respectively, using; the three shims (**A**, **B**), Five different pixel sizes (**C**, **D**), three slice thickness (**E**, **F**) and five TR values (**G**, **H**).

**Figure 2:**
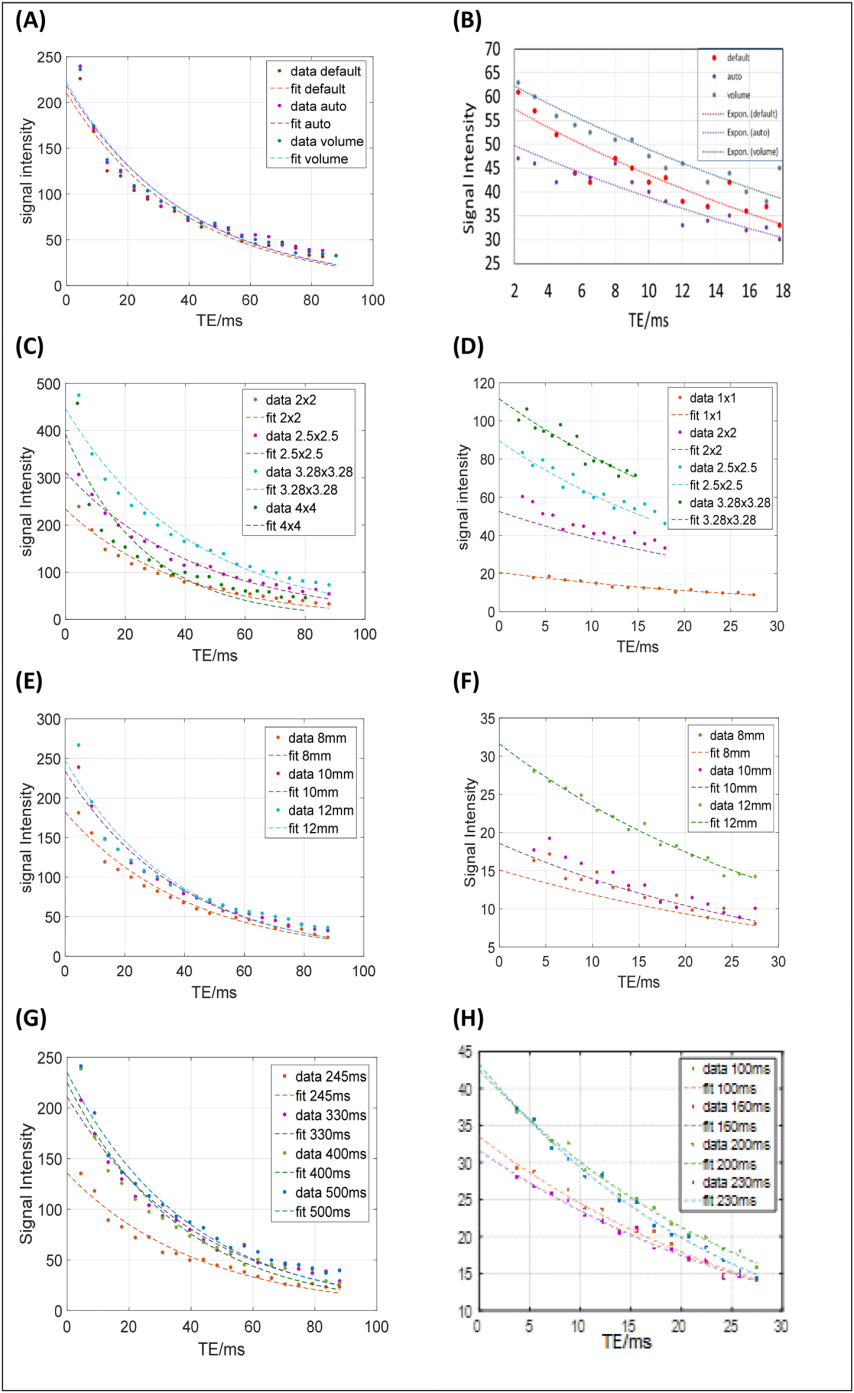
Decay curves of T2 and T2* for a healthy volunteer using: the three shim techniques (**A**, **B**), pixel sizes of 2 × 2 to 4 × 4 mm for T2 (**C**), pixel sizes of 1 × 1 to 3.28 × 3.28 mm for T2* (**D**), slice thicknesses of 8, 10, 12 mm (**E**, **F**), TR values 245–500 ms for T2 (**G**) and TR values 100–230 ms for T2* (**H**).

**Figure 3:**
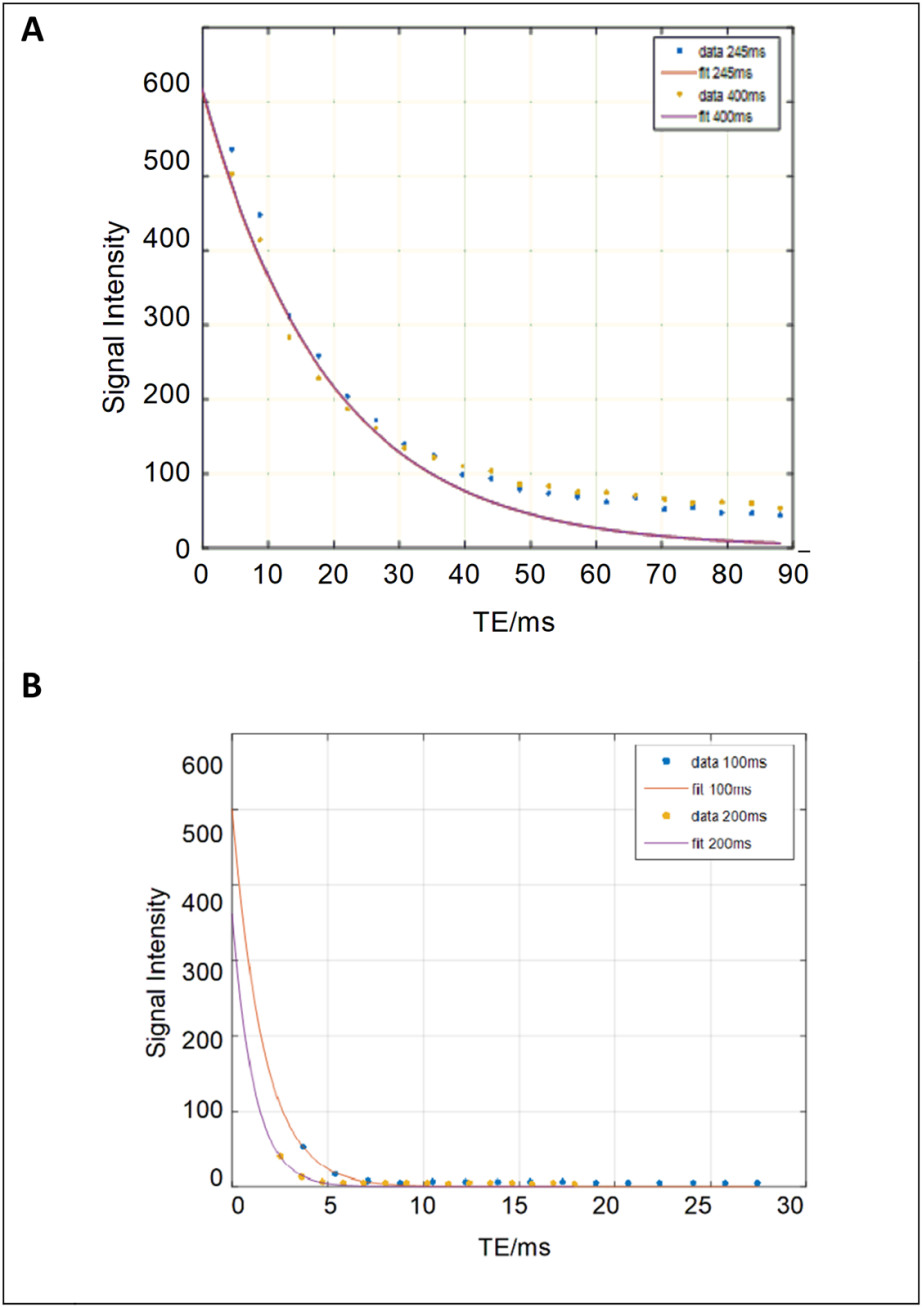
Decay curve of a sickle cell patient: (**A**) using auto shim, 2 × 2 mm pixel size, 10 mm slice thickness, and TR = 245 ms for T2 sequence ; (**B**) at default shim, 1 × 1 mm pixel size, 12 mm slice thickness, and TR = 100 ms for T2* sequence.

**Figure 4:**
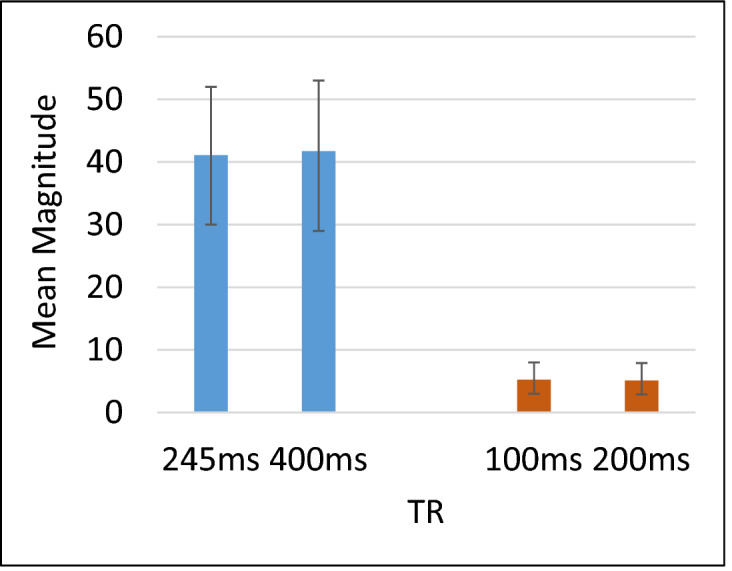
Mean magnitude measurements (with SD) of the 25 patients using the conventional T2 and T2* sequences versus the new optimised sequences.

**Figure 5:**
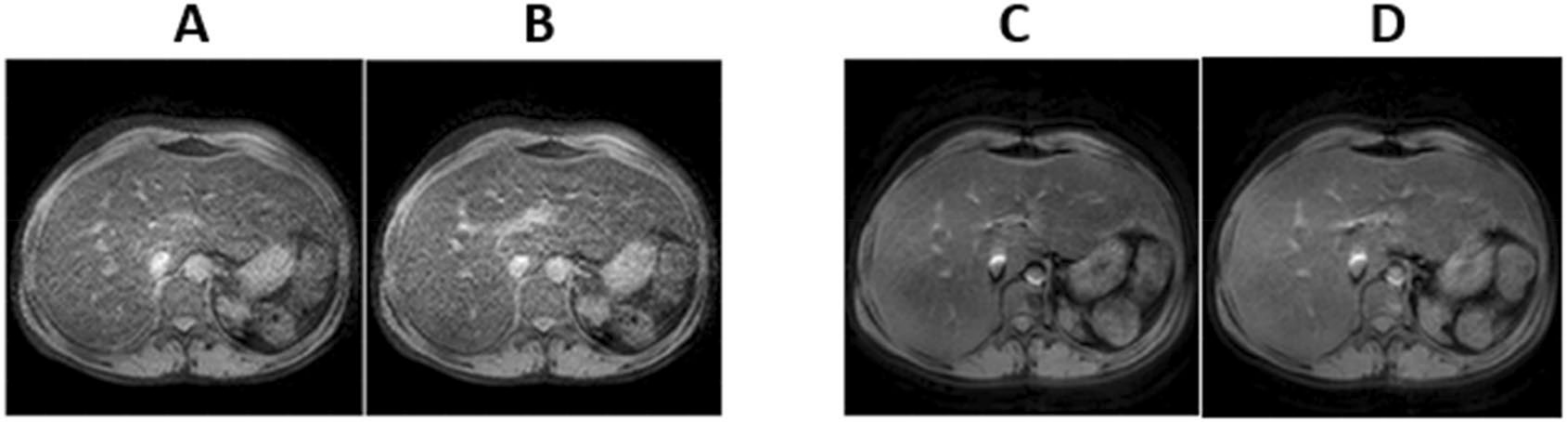
MRI images of the same patient using: (**A**) standard T2*, (**B**) optimised T2*, (**C**) standard T2 and (**D**) optimised T2 sequences.

The effects of tested parameters were studied in terms of three performance indices: (i) The exponential decay curves of both T2 and T2*; (ii) the magnitude measurements of T2 or T2* values using Eq. 1 and (iii) the correlation coefficient R^2^, which determines the associated strength between two variables. All results are presented in in Figs. 1, 2, 3, 4 and 5 and Tables 1 and 2.

**Table 1:** Phantom mean measurements ± standard deviation of T2 and T2* for different shim techniques.

Sequence	Shim type	1%	2%	3%	4%	5%	20%
T2	Default	49.36 ± 0.79	26.01 ± 0.33	17.43 ± 0.20	13.70 ± 0.15	11.27 ± 0.14	5.58 ± 1.49
	Auto	49.57 ± 0.14	26.19 ± 0.11	17.52 ± 0.17	13.78 ± 0.13	11.29 ± 0.13	5.66 ± 0.97
	Volume	49.58 ± 0.46	25.91 ± 0.58	17.54 ± 0.26	13.75 ± 0.21	11.21 ± 0.20	6.06 ± 1.27
T2*	Default	34.90 ± 2.81	20.04 ± 0.25	14.07 ± 0.33	11.21 ± 0.39	8.08 ± 0.29	1.80 ± 0.09
	Auto	28.62 ± 2.83	19.21 ± 0.22	13.65 ± 0.32	11.02 ± 0.23	7.80 ± 0.26	1.70 ± 0.09
	Volume	30.62 ± 3.45	19.28 ± 0.24	13.88 ± 0.33	11.07 ± 0.37	7.83 ± 0.26	1.72 ± 0.09

**Table 2:** Phantom mean measurements ± standard deviation for different pixel size using T2 and T2*.

Sequence	Voxel size	1%	2%	3%	4%	5%	20%
T2	1 × 1	47.25 ± 0.09	24.30 ± 0.1	16.33 ± 0.14	12.61 ± 0.10	10.11 ± 0.141	4.79 ± 0.80
	2 × 2	50.57 ± 0.14	26.19 ± 0.11	17.52 ± 0.17	13.78 ± 0.13	11.29 ± 0.132	5.72 ± 0.97
	2.5 × 2.5	53.28 ± 2.73	27.66 ± 0.32	18.81 ± 0.60	14.38 ± 0.46	11.71 ± 0.494	6.00 ± 0.99
	3.28 × 3.28	54.23 ± 3.64	27.84 ± 0.31	19.17 ± 0.79	15.19 ± 0.62	12.86 ± 0.589	7.15 ± 0.95
	4 × 4	54.84 ± 4.07	27.99 ± 0.39	19.57 ± 0.74	15.88 ± 0.60	12.73 ± 0.572	7.29 ± 1.00
T2*	1 × 1	36.74 ± 0.91	21.18 ± 0.31	15.25 ± 0.06	12.01 ± 0.20	9.60 ± 0.16	1.75 ± 0.12
	2 × 2	44.10 ± 1.76	23.72 ± 0.69	18.52 ± 0.29	13.70 ± 0.36	10.28 ± 0.38	2.23 ± 0.12
	2.5 × 2.5	45.88 ± 3.51	24.21 ± 0.36	19.99 ± 0.36	13.89 ± 0.34	11.12 ± 0.48	2.01 ± 0.12
	3.28 × 3.28	50.72 ± 7.78	22.47 ± 0.50	20.13 ± 0.40	13.99 ± 0.45	11.16 ± 0.49	2.08 ± 0.20
	4 × 4	50.03 ± 11.16	20.82 ± 1.54	20.22 ± 0.52	14.00 ± 0.44	11.56 ± 0.53	2.21 ± 0.37

#### Phantom and volunteers results

The results of the phantom and volunteers measurements, are presented in Tables 1 and 2 and in Figs. 1 and 2. The figures show plots of the 2% Gd concentration versus echo time (TE) for three shims, five different pixel sizes, three slice thickness and five TR values. The tables show mean magnitude measurements of Gd concentrations 1–20% and of 10 volunteers respectively, for both T2 and T2*, using the tested parameters.

#### Patients results

The results of serum ferritin for the volunteers ranged between 5.5 and 86.8 ng/ml. For the patients, the serum measurements ranged between 179 and 2000 ng/ml. T2 decay curves of standard (TR 400 ms) versus optimised (TR 245 ms) of a sickle cell patient with iron overload are shown in Fig. 3a. Figure 3b shows the decay curves of the standard (TR = 200 ms) versus optimised (TR = 100 ms) for T2* for the same patient. The magnitude measurements of the 25 patients using the conventional T2 and T2* sequences versus the new optimised sequences are shown in Fig. 4.

The mean magnitudes of the standard and optimised sequences for T2 were 41.72 ± 11.82 ms and 41.10 ± 11.31 ms, and for T2* they were 5.11 ± 3.39 and 5.25 ± 3.49 ms respectively.

Figure 5A,B present typical images of a patient acquired using standard and optimised T2*, while Fig. 5C,D obtained using standard and optimised T2, respectively.

## Discussion

It was observed that all decay curves of the phantom studies appeared with offset at the first two data points of the curve. The T2* curves experience slight oscillation. The presence of field inhomogeneities probably caused this oscillation. In Fig. 1 the three shim techniques of T2 and T2* sequences appeared overlapping, indicating that there is no significant variation between their measurements. Increasing the pixel size (in- resolution) caused an increase in the bandwidth (the scanner was not set for a fixed Hz/pixel value). Nevertheless, with the increase in bandwidth, the signal-to-noise ratio was reduced simultaneously. The oscillation of data points around the curve was noticed to increase with higher Gd concentrations. Slice thickness had a minor effect on the T2 measures, while the signal intensity was noticed to increase with slice thickness for T2*. TR was observed to have a minor effect on both sequence measurements.

Results for volunteers, as shown in Fig. 2, indicate that T2 and T2* were loosely fit to its associated decay curves. Factors such as blood, the anatomy of the organ may have contributed to poor fit. However, T2 curves are bound together, indicating that shim has no significant effect on the measurements. Concurrently, T2* data points exhibited higher oscillation than those of the T2, which is attributed to the presence of field inhomogeneities. Such small oscillation can likely have profound effects on estimating long T2* values^30^. The scattering of data points were noticed to be specifically higher at pixel sizes of 3.28 × 3.28 and 4 × 4 mm, especially for T2* measurements. The trend of the decay curves for the different slice thickness for T2 appeared the same. However, the signal intensity measurements for T2* were noticed to be the highest at 12 mm.

Tables 1, 2, 3 and 4 represent the magnitude measurements (with SD) of Gd concentrations of 1–20% for the different shim techniques, pixel sizes, slice thicknesses, and TR values for both T2 and T2* measurements. Generally, it can be concluded that there is no significant differences in measurements for the three shim techniques. Statistical test showed correlations coefficients (R^2^) ranging from 0.98 to 0.99 for the three shim techniques. However, the 1% Gd concentration experienced different T2* measurements since it recorded the highest magnitude measurement with the lowest (SD). On the other hand, the increase in pixel sizes resulted in a steady increase in magnitude measurements. The 20% concentration recorded the lowest R^2^ and highest SD, especially at smaller pixel sizes. This is probably because low T2 measurements cannot be measured accurately due to the limitation of the short echo time and echo spacing used (5.9/5.9 ms). For T2* measurements, the 1 × 1 mm recorded the lowest magnitude measurements with low SD for the whole range of Gd concentrations. Measurements fluctuations can be observed at 4–20% Gd concentrations and the magnitude values increased by nearly twice as much when using pixel sizes 3.28 × 3.28 mm or 4 × 4 mm compared to the 1 × 1 mm pixel. R^2^ values ranged between 0.514 and 0.997 for all Gd concentrations using these pixel sizes, with the optimum obtained at 1 × 1 mm.

**Table 3:** Phantom mean measurements ± standard deviation of different slice thickness for T2 and T2*.

Sequence	Slice thickness (mm)	1%	2%	3%	4%	5%	20%
T2	8	49.96 ± 0.42	27.24 ± 0.17	17.32 ± 0.49	13.01 ± 0.26	11.12 ± 0.15	5.40 ± 0.79
	10	52.57 ± 0.09	27.30 ± 0.17	17.47 ± 0.44	13.61 ± 0.20	11.18 ± 0.14	5.22 ± 0.70
	12	52.97 ± 0.26	27.19 ± 0.17	17.40 ± 0.45	13.67 ± 0.21	11.20 ± 0.15	4.88 ± 0.71
T2*	8	41.51 ± 0.70	24.05 ± 0.25	15.27 ± 0.18	12.36 ± 0.22	9.68 ± 0.04	2.42 ± 0.06
	10	36.74 ± 0.51	22.18 ± 0.31	15.25 ± 0.06	12.01 ± 0.20	9.60 ± 0.03	1.77 ± 0.12
	12	31.63 ± 0.44	22.13 ± 0.30	15.24 ± 0.06	12.02 ± 0.05	9.36 ± 0.02	1.75 ± 0.01

**Table 4:** Phantom mean measurements ± standard deviation of different TR using T2 and T2* sequences.

Sequence	TR	1%	2%	3%	4%	5%	20%
T2	TR 212	49.47 ± 0.48	25.32 ± 0.11	16.56 ± 0.22	12.78 ± 0.07	10.06 ± 0.13	5.99 ± 0.33
	TR 245	49.83 ± 0.55	25.51 ± 0.11	16.72 ± 0.22	12.84 ± 0.06	10.10 ± 0.12	4.83 ± 0.40
	TR 330	49.88 ± 0.23	25.73 ± 0.35	17.07 ± 0.31	13.06 ± 0.23	10.10 ± 0.13	3.99 ± 0.58
	TR 400	50.09 ± 0.19	26.50 ± 0.40	17.49 ± 0.32	13.50 ± 0.20	10.84 ± 0.14	4.72 ± 0.87
	TR 500	50.15 ± 0.19	26.67 ± 0.40	17.66 ± 0.34	13.51 ± 0.29	10.84 ± 0.15	3.91 ± 1.41
T2*	TR 100	40.52 ± 0.22	19.45 ± 0.12	13.05 ± 0.10	9.82 ± 0.04	8.26 ± 0.04	1.61 ± 0.09
	TR 160	40.66 ± 0.28	19.69 ± 0.14	13.07 ± 0.18	9.72 ± 0.11	8.19 ± 0.05	1.66 ± 0.09
	TR200	40.68 ± 0.34	19.73 ± 0.10	13.24 ± 0.06	9.72 ± 0.05	8.36 ± 0.02	1.75 ± 0.01
	TR 230	40.72 ± 0.22	19.66 ± 0.19	13.38 ± 0.12	9.78 ± 0.11	8.13 ± 0.04	1.63 ± 0.13
	TR 300	40.86 ± 0.34	19.70 ± 0.25	13.31 ± 0.13	9.74 ± 0.11	8.15 ± 0.12	1.82 ± 0.11

The slice thickness study on the 1% and 2% Gd using T2* sequence demonstrated higher magnitude measurements at 8 mm, while the 12 mm thickness showed low SD for all the concentrations with R^2^ ranging 0.975–0.997at p < 0.01. Moreover, TR values had small effects on T2 measurements while 1–2% Gd concentration demonstrated a slight increase in measurements with TR. It was also observed that TR has a minor effect on T2* measurements with the lowest fluctuations of measurements found for concentrations of 3–20% with R^2^ range between 0.98 and 0.996.

Tables 5 and 6 show mean ± SD of the result of magnitude measurements calculated on volunteers for both T2 and T2* sequences. Average T2 measurements on normal volunteers' livers using the T2 sequence in this study was 44 ± 7 ms comparable with other similar studies^31–33^. We also observed that the average magnitude measurement using the default shim was less than the measurements recorded with auto and volume shim techniques by approximately 0.1 and 0.2%, respectively. The default shim recorded high SD values, while the low fluctuation of measurements was recorded using the auto-shim technique. The overall T2* measurements recorded from the volunteers fell between 30 and 45 ms (average 36 ± 5 ms) comparable with literature^34^. The T2 magnitude measurements were presented with a slight increase for pixel sizes larger than 2.5 × 2.5 mm.

**Table 5:** Volunteers measurement of magnitude ± standard deviation for T2 and T2* sequences.

	T2	T2*
**Shim**		
Default	44.09 ± 7.09	36.87 ± 11.93
Auto	44.73 ± 6.25	35.48 ± 12.36
Volume	45.12 ± 6.76	36.37 ± 12.17
**Pixel sizes**		
1 × 1	–	34.10 ± 7.71
2 × 2	44.09 ± 6.07	36.87 ± 11.60
2.5 × 2.5	46.86 ± 9.29	37.15 ± 12.93
3.28 × 3.28	47.60 ± 9.66	39.01 ± 17.87
4 × 4	47.63 ± 9.86	–
**Thicknesses**		
8 mm	43.33 ± 7.26	34.92 ± 11.48
10 mm	44.09 ± 6.07	34.54 ± 7.71
12 mm	44.09 ± 6.45	36.41 ± 5.91

**Table 6:** Volunteers measurement of magnitude ± standard deviation of the ten volunteers using different TR values for both T2 and T2* sequences.

TR	T2	TR	T2*
TR245	45.65 ± 6.62	TR100	33.84 ± 7.44
TR330	46.66 ± 6.41	TR160	34.14 ± 7.20
TR400	46.09 ± 6.52	TR200	36.41 ± 5.91
TR500	47.39 ± 4.95	TR230	36.58 ± 5.84

The T2* magnitude measurements at different pixel sizes showed similarity to the magnitude measurement obtained for the 1% Gd concentration. The lowest measurement was recorded at 1 × 1 mm. Larger pixel sizes provided T2* measurements of up-to 20% than that of the 1 × 1 mm. It is also observed that the SD values became higher with pixel size (reaching up to 50% for pixel size of 4 × 4 mm).

In comparison with the literature, our study found that the increase of pixel size resulted in an increase in the sensitivity of the magnitude measurements^35^. Larger pixel sizes have caused the measurements to become less reliable. Similar to the T2 measurements the utilization of higher receiver bandwidth in this study incorporated more noise on the acquired images, and subsequently caused scattering in signal intensity measurements. This led to wide fluctuations of measurements especially in the case of high pixel sizes of 3.28 × 3.28 mm and 4 × 4 mm (R^2^ ~ 0.614). The T2 sequence results showed no significant differences between the magnitude measurements for the slice thicknesses of 8, 10, and 12 mm. As for T2*, the 12 mm thickness was associated with the highest magnitude measurements and lowest fluctuations of the measurements.

Mean magnitude measurements of T2 for the range of studied TR witnessed no significant difference in their values. T2* measurements at TR values of 100-160 ms, recorded low mean magnitude of measurements (by ~ 30%) compare to those at 200-230 ms. Long T2* components, like those found in healthy volunteers, require long TR values for accurate measurements. It is observed that long TRs of 200–230 ms provides consistent results (R^2^ range 0.95–0.99).

In general, there was a slight difference in magnitude measurements between the conventional and optimised T2 sequences for patients studies (Figs. 3, 4). The conventional sequence provided a higher measurement by 1.5% and higher SD by 4% than its corresponding optimised sequence. The correlation coefficients obtained from the conventional sequence were 0.978–0.994, whilst for the optimised sequence the range was 0.979–0.994 (p < 0.01). We also observed similar image qualities were obtained for the patients using the T2 and T2* optimised sequences as compared to their corresponding standard sequences (Fig. 5).

Thus, the results indicate that the standard T2 sequence could easily replace the optimised sequence without compromising sickle cell patients' measured data with iron overload. Similarly results obtained for T2* sequences were in close agreement with this conclusion (with uncertainty of ~ 3%). The correlation coefficient for the standard sequence ranged between 0.942–0.99, while for the optimised (0.945–1.0) at p-value < 0.01.

Our studies on the effects of these parameters were for the first time investigated on the livers of transfusion-dependent paediatric patients using both T2 and T2* sequence. Our investigations showed a room for further improvement in the T2* sequence by adopting the optimised sequence instead of the conventional sequence. Another advantage of this optimisation is that the acquisition time for the T2 sequence was reduced by almost 38% and for T2* by 25%. We also noticed that the measurements' reliability has dramatically improved.

This study has some limitations to generalize its outcomes for a number of reasons: (i) the number of samples is relatively small (10 volunteers + 25 patients). (ii) To get the optimum pixel size and keep the Hz/pixel fixed, this requires a change in both the field of view (FOV) and the matrix size. This type of modification affects the routine of work in the hospital so we had to keep the FOV and the size of the matrix constant throughout the study period.

## Conclusion

The aim of this study was to investigate the effect of different shim techniques, voxel sizes, and repetition time (TR) on T2 and T2* sequences and to determine optimum settings for the evaluation of the amount of iron in the livers of transfusion dependent sickle cell patients. Phantom with different concentrations of Gd (1–20%), and ten volunteers were used to define these settings, which were then investigated on 25 patients. The optimized protocols resulted in a reduction of acquisition time by 38% and 25% for T2 and T2*, respectively. In addition, the quality of images were not compromised as compared to the conventional sequences. The outcomes of this study could be particularly beneficial for young adults who cannot tolerate MRI scans for long periods of time. The research results indicated that T2 and T2* sequences can be further refined to provide more reliable measurements and on transfusion dependent-sickle cell patients.
