# Author Correction: Optimisation of T2 and T2* sequences in MRI for better quantification of iron on transfused dependent sickle cell patients

**DOI:** 10.1038/s41598-021-96977-2

**Published:** 2021-08-26

**Authors:** Azza Ahmed, Amani Baldo, A. Sulieman, Hind Mirghani, Fouad A. Abolaban, I. I. Suliman, Isam Salih

**Affiliations:** 1 Sudan Atomic Energy Commission, Al Gamah Street, P. O. Box 3001, Khartoum, Sudan; 2 Jaffar Bin Auf Paediatric Hospital, Hosptials Road, Khartoum, Sudan; 3 Radiology and Medical Imaging Department, College of Applied Medical Sciences, Prince Sattam Bin Abdulaziz University, P. O. Box 422, Alkharj, 11942 Saudi Arabia; 4 Nuclear Engineering Department, Faculty of Engineering, King Abdulaziz University, P. O. Box 80221, Jeddah, 21589 Saudi Arabia; 5 Physics Department, College of Science, Imam Mohammad Ibn Saud Islamic University (IMSIU), P. O. Box 11642, Riyadh, Saudi Arabia; 6 Basic Science Department, Prince Sattam Bin Abdulaziz University, P. O. Box 422, Alkharj, 11942 Saudi Arabia

Correction to: *Scientific Reports* 10.1038/s41598-021-88116-8, published online 19 April 2021

The original version of this Article contained an error in Affiliation 5, which was incorrectly given as ‘Physics Department, College of Science, Imam Mohamed Ibn Saud Islamic University (IMSIU), P. O. Box 11642, Riyadh, Saudi Arabia’. The correct affiliation is listed below:

Physics Department, College of Science, Imam Mohammad Ibn Saud Islamic University (IMSIU), P. O. Box 11642, Riyadh, Saudi Arabia

The original Article has been corrected.

